# CD8^+^ T cells specific for conserved, cross-reactive Gag epitopes with strong ability to suppress HIV-1 replication

**DOI:** 10.1186/s12977-018-0429-y

**Published:** 2018-07-03

**Authors:** Hayato Murakoshi, Chengcheng Zou, Nozomi Kuse, Tomohiro Akahoshi, Takayuki Chikata, Hiroyuki Gatanaga, Shinichi Oka, Tomáš Hanke, Masafumi Takiguchi

**Affiliations:** 1 Center for AIDS Research, Kumamoto University, 2-2-1 Honjo, Chuo-ku, Kumamoto, 860-0811 Japan; 2 International Research Center of Medical Sciences, Kumamoto University, Kumamoto, Japan; 3 AIDS Clinical Center, National Center for Global Health and Medicine, Tokyo, Japan; 4 The Jenner Institute, University of Oxford, Old Road Campus Research Building, Roosevelt Drive, Oxford, UK

**Keywords:** HIV-1, Gag, CTL, Vaccine, Conserved epitope

## Abstract

**Background:**

Development of AIDS vaccines for effective prevention of circulating HIV-1 is required, but no trial has demonstrated definitive effects on the prevention. Several recent T-cell vaccine trials showed no protection against HIV-1 acquisition although the vaccines induced HIV-1-specific T-cell responses, suggesting that the vaccine-induced T cells have insufficient capacities to suppress HIV-1 replication and/or cross-recognize circulating HIV-1. Therefore, it is necessary to develop T-cell vaccines that elicit T cells recognizing shared protective epitopes with strong ability to suppress HIV-1. We recently designed T-cell mosaic vaccine immunogens tHIVconsvX composed of 6 conserved Gag and Pol regions and demonstrated that the T-cell responses to peptides derived from the vaccine immunogens were significantly associated with lower plasma viral load (pVL) and higher CD4^+^ T-cell count (CD4 count) in HIV-1-infected, treatment-naive Japanese individuals. However, it remains unknown T cells of which specificities have the ability to suppress HIV-1 replication. In the present study, we sought to identify more T cells specific for protective Gag epitopes in the vaccine immunogens, and analyze their abilities to suppress HIV-1 replication and recognize epitope variants in circulating HIV-1.

**Results:**

We determined 17 optimal Gag epitopes and their HLA restriction, and found that T-cell responses to 9 were associated significantly with lower pVL and/or higher CD4 count. T-cells recognizing 5 of these Gag peptides remained associated with good clinical outcome in 221 HIV-1-infected individuals even when comparing responders and non-responders with the same restricting HLA alleles. Although it was known previously that T cells specific for 3 of these protective epitopes had strong abilities to suppress HIV-1 replication in vivo, here we demonstrated equivalent abilities for the 2 novel epitopes. Furthermore, T cells against all 5 Gag epitopes cross-recognized variants in majority of circulating HIV-1.

**Conclusions:**

We demonstrated that T cells specific for 5 Gag conserved epitopes in the tHIVconsvX have ability to suppress replication of circulating HIV-1 in HIV-1-infected individuals. Therefore, the tHIVconsvX vaccines have the right specificity to contribute to prevention of HIV-1 infection and eradication of latently infected cells following HIV-1 reactivation.

**Electronic supplementary material:**

The online version of this article (10.1186/s12977-018-0429-y) contains supplementary material, which is available to authorized users.

## Background

Development of effective vaccines against HIV-1 is the best hope for controlling the AIDS epidemic, but no trials has yet showed definitive effect on prevention of HIV-1 infection. Although the RV144 trial in Thailand showed weak protection against HIV-1 most likely through generation of non-neutralizing antibodies [1–7], the outcome of this vaccine trial remains to be reproduced [8, 9]. The STEP study of a candidate T-cell vaccine induced low frequency responses in 77% of vaccine recipients and showed no protection against HIV-1 acquisition [10, 11]. Although a sieve effect of break-through viruses was described, overall, the vaccine-elicited CD8^+^ T cells had insufficient capacity to suppress HIV-1 replication. There is no simple functional or phenotypic T-cell marker consistently associated with HIV-1 control. The protective capacity of CD8^+^ T cells likely comes from multiple attributes including specificity, breadth, quality, quantity and being at the right time at the right place. Targeting protective epitopes is one of the key traits.

Our strategy for induction of effective responses is to focus the CD8^+^ T cells on the highly functionally conserved regions of the HIV-1 proteome [12, 13]. To further improve their efficacy, vaccine immunogens tHIVconsvX consist of 6 protein regions from Gag and Pol with high coverage of known protective epitopes and employ a bi-valent mosaic (two versions of each region, which differ in approximately 1/10 amino acids) to maximize the match of the vaccine to the global circulating viruses. Indeed, using overlapping 15-mer peptides derived from the tHIVconsvX immunogens, we demonstrated the correlation of both the total magnitude and breadth of the tHIVconsvX-specific T-cell responses to lower plasma viral load (pVL) and higher CD4^+^ T-cell count (CD4 count) in a cohort of 120 treatment-naïve, HIV-1-positive patients in Japan [14].

Numerous studies have showed that CD8^+^ T cell targeting of HIV-1 Gag linked to viral load or disease outcome in HIV-1 infection [15–17]. The mechanism of this effect may involve that the viral genome is delivered into the cell in a ribonucleoprotein complex composed largely of Gag after infection, so that Gag epitopes can be processed, presented on the cell surface and finally recognized by Gag-specific CD8^+^ T cells within a few hours of infection before Nef-mediated down-regulation takes place [18–20].

We previously showed association of CD8^+^ T cells specific for several Gag and Pol epitopes with significantly lower pVL and higher CD4 count in chronically HIV-1-infected Japanese patients [21]. The majority of these CD8^+^ T cells recognized conserved or cross-recognized mutated epitopes mostly on the Gag protein. Half of these T cells were restricted by 2 protective alleles, HLA-B*52:01 or HLA-B*67:01, while the remaining recognized peptides presented by HLA-A*02:06, HLA-B*40:02, or HLA-B*40:06.

In the present study, we determined fine specificities and HLA-restriction of CD8^+^ T-cell responses specific for the two Gag conserved regions of the candidate tHIVconsvX vaccine and extended the protective correlations to clinical outcome in 221 treatment-naïve HIV-1-positive patients. The study identified additional CD8^+^ T cells specific for Gag conserved epitopes with strong ability to suppress HIV-1 replication. Thus, Gag-specific CD8^+^ T cells induced by the tHIVconsvX vaccine have the potential to significantly contribute to prevention of HIV-1 infection and eradication of latently infected cells.

## Results

### T-cell responses to the conserved regions of Gag were protective

**Fig. 1:**
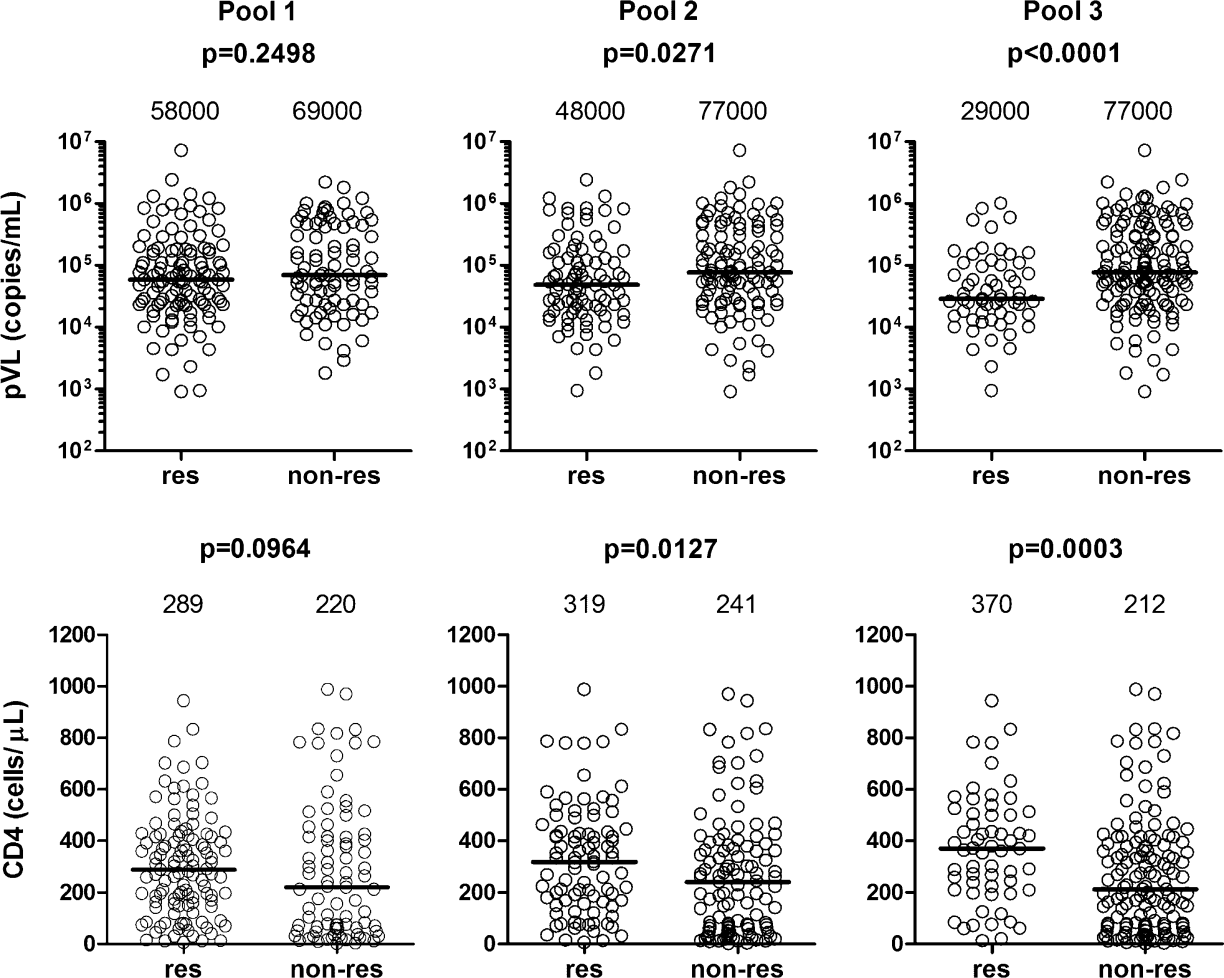
Correlation of T-cell responses to Gag conserved peptides of tHIVconsvX. Correlation of T-cell responses to Gag conserved peptides of tHIVconsvX with pVL and CD4 count. T-cell responses to Gag peptide Pools 1, 2 and 3 derived from vaccine immunogen tHIVconsvX were enumerated using an IFN-γ ELISPOT assay in 200 HIV-1-infected Japanese individuals. Comparison of pVL and CD4 count between responders and non-responders to the Gag peptides was statistically analyzed using the Mann-Whitney test. The value in each figure represents the median of pVL and CD4 count

We previously showed in 120 treatment-naive HIV-1^+^ Japanese patients that CD8^+^ T-cell responses specific for the conserved regions of the tHIVconsvX immunogen were protective [14]. Here, we focused on the two vaccine conserved regions of Gag, since the responses to Gag had the strongest effect on the suppression of HIV-1 replication in most previous studies [15–17]. We analyzed additional 80 treatment-naive HIV-1-positive patients and reanalyzed the data together from 200 individuals for the magnitude and breadth of CD8^+^ T-cell responses to 3 pools of the Gag 15-mer peptides (Pools 1, 2, and 3). The magnitude and breadth of the T-cell responses to Pools 2 and 3, and particularly responses to Pool 3, were significantly correlated with high CD4 count and low pVL, whereas those to Pool 1 were not (Fig. 1 and Additional file 1: Fig. S1). These results suggest that T cells specific for peptides within Pools 2 and 3 contribute to the suppression of HIV-1 replication.

### Mapping the CD8^+^ T-cell Gag-specificity to optimal epitopes

**Fig. 2:**
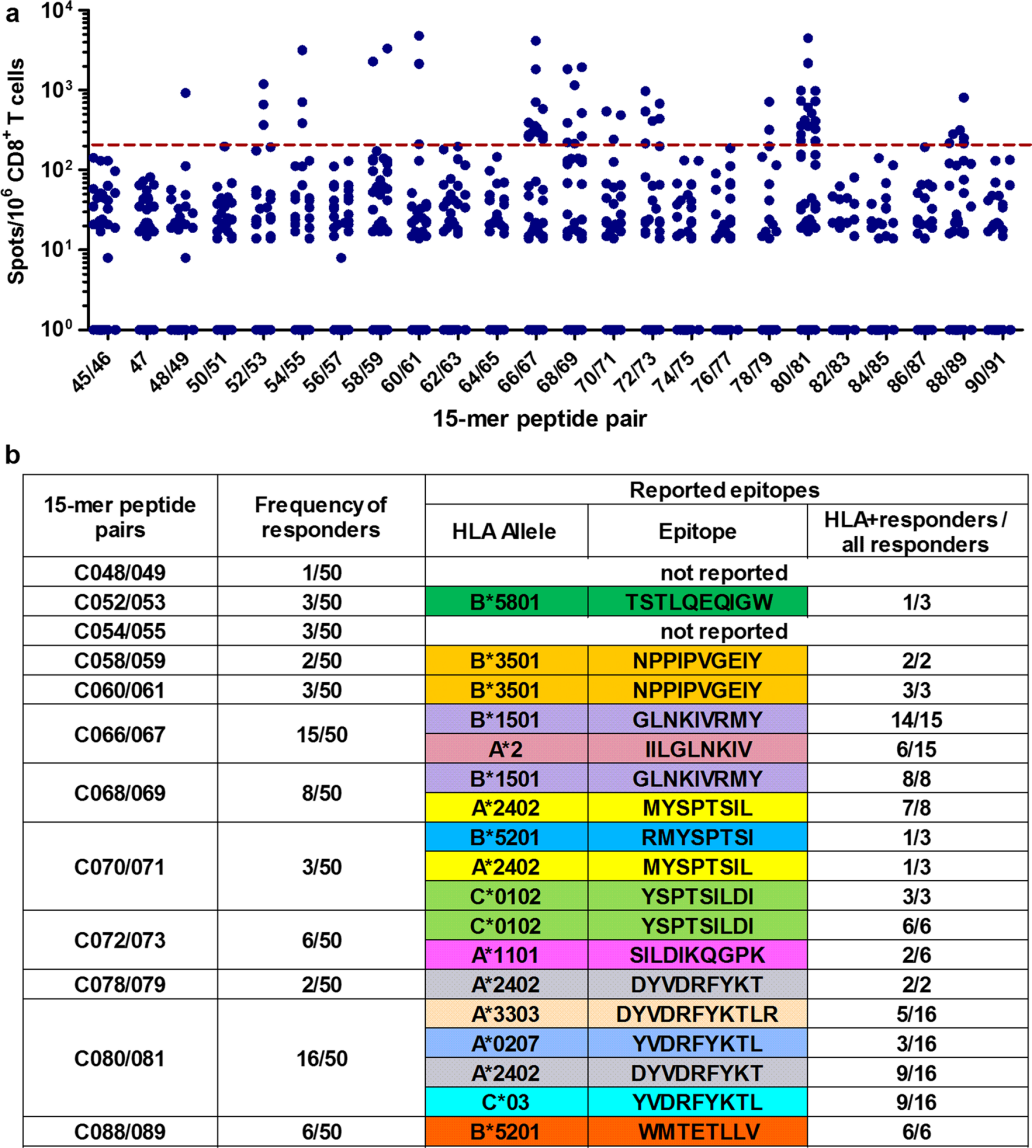
T-cell responses to 15-mer peptide pairs in Gag Pool 2. **a** The responses to individual pairs of 15-mer peptides in Gag Pool 2. Responses to peptide pairs in 50 responders to Pool 2 were analyzed by an IFN-γ ELISPOT assay. The dotted line at 200 SFU/10^6^ CD8^+^ T cells indicates a threshold for a positive response. **b** Summary of responders and peptide pairs. Reported epitopes present in the pair of the 15-mer peptides according to the LANL-HSD. HLA^+^ responders are those with the matching restricting HLA allele for the reported epitope

**Fig. 3:**
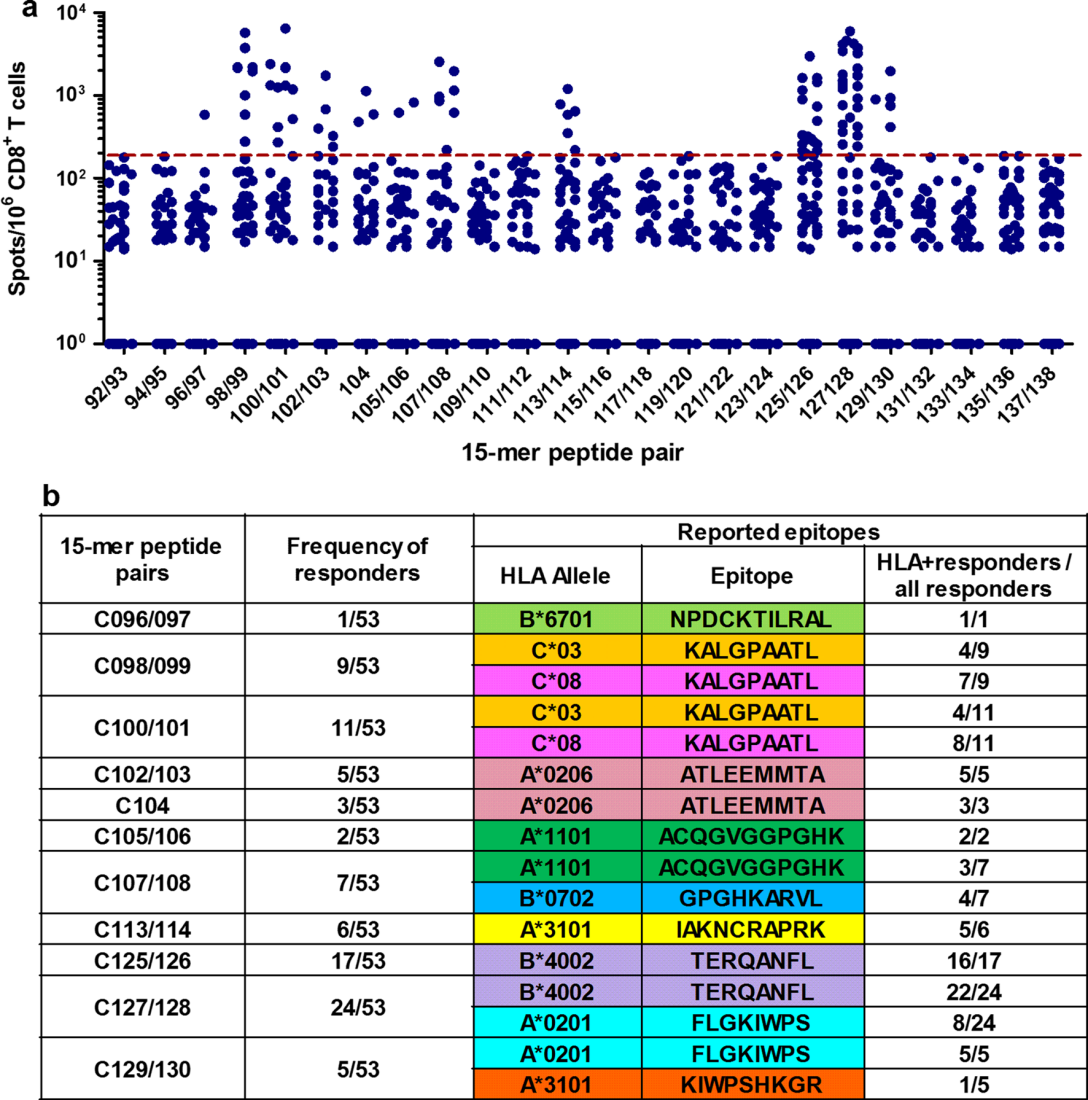
T-cell responses to 15-mer peptide pairs in Gag Pool 3. **a** The responses to individual pairs of 15-mer peptides in Gag Pool 3. Responses to peptide pairs in 53 responders to Pool 3 were analyzed by an IFN-γ ELISPOT assay. The dotted line at 200 SFU/10^6^ CD8^+^ T cells indicates a threshold for a positive response. **b** Summary of responders and peptide pairs. Reported epitopes included in the pair of the 15-mer peptides are shown according to the LANL-HSD. HLA^+^ responders are those with the matching restricting HLA allele for the reported epitope

We found that 89 and 59 individuals recognized 15-mer peptides in Pools 2 and 3, respectively. Of these, we selected 50 and 53 based on sufficient PBMC sample availability for the fine definition of optimal epitopes. Pools 2 and 3 included peptide pairs derived from the bi-valent mosaic and one single peptide common between the two mosaics. By using an IFN-γ ELISPOT assay, we found T-cell responses to 12 peptide pairs in Pool 2 (Fig. 2a) and 10 peptide pairs and one common peptide in Pool 3 (Fig. 3a) in at least one individual. These 15-mer peptides contained sequences of previously reported epitopes; 13 epitopes in Pool 2 and 10 in Pool 3 (Figs. 2b and 3b). Upon inspection of the subjects’ HLA, most of the responders had the reported restricting alleles (Figs. 2b and 3b). However, responders to 15-mer peptides C48/49, C052/053, C054/055, C113/114, or C125/126 did not have the matching restricting HLA alleles, indicating that their CD8^+^ T cells may recognize novel, previously unreported epitopes.

**Fig. 4:**
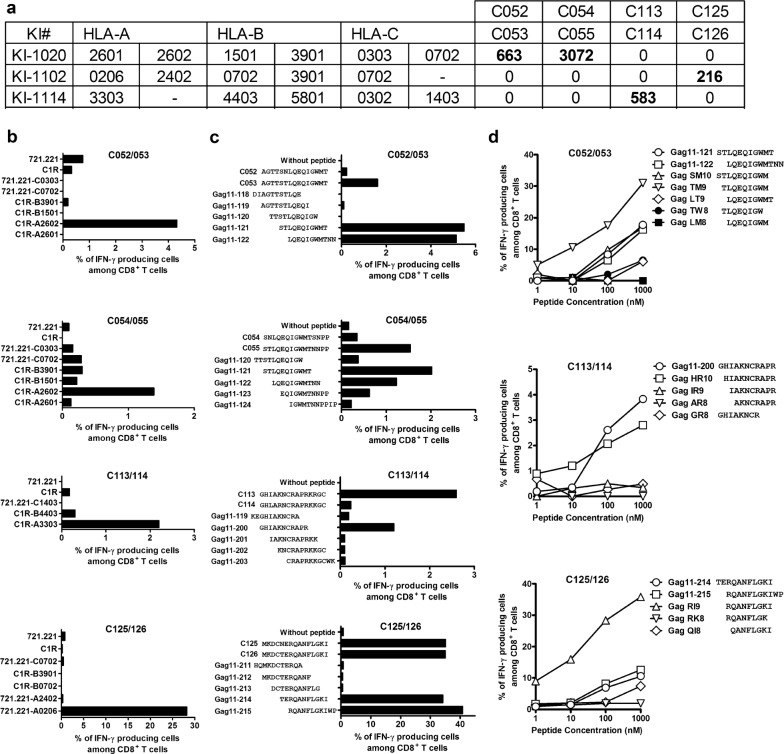
Identification of novel T-cell Gag epitopes. **a** T-cell responses to peptide pairs in individuals KI-1020, KI-1102, and KI-1114, who did not have matching HLA alleles for previously reported epitopes. **b** HLA restriction. Responses by STCL stimulated with C1R or 721.221 cells expressing individual HLA molecule shared by the responders and pulsed with the peptide pair were analyzed by ICS assay. C052/053 and C054/055 peptides were analyzed by using the T cells derived from KI-1020, while C113/114 and C125/126 by using the T cells from KI-1114 and KI-1102, respectively. **c** Identification of 11-mer HIV-1 clade B overlapping peptides recognized by the HLA-A*26:02-, HLA-A*33:03-, or HLA-A*02:06-restricted T cells. The responses by STCL expanded with C052/053, C054/055, C113/114, or C125/126 peptide pairs to the corresponding stimulator cells pre-pulsed with 11-mer HIV-1 clade B-derived overlapping peptides across the parental 15-mer were analyzed in ICS assay. **d** Identification of optimal peptides. The STCL responses stimulated with peptide pairs C052/053, C113/114, or C125/126 to the corresponding stimulator cells C1R-A2602, C1R-A3303, or 721.221-A0206 pre-pulsed with individual truncated peptides were analyzed by ICS assay

Next, we sought to define these novel epitopes and their HLA restriction. We did not pursue the epitope in C48/49 since only one responder to this peptide pair was detected in the 50 tested individuals. The responder KI-1020 had T cells specific for the C052/053 and C054/055 peptide pairs, while KI-1102 and KI-1114 had responses to C125/126 and C113/114 peptides, respectively (Fig. 4a). To define the HLA restriction, subjects’ PBMCs were first expanded with each peptide pair for 12–14 days and these STCL were tested in ICS assay using either C1R or 721.221 cells stably transfected with all subject’s HLA class I molecules and pulsed with the peptides. The results showed that both T-cell responses to the C052/053 and C054/055 15-mer peptide pairs were restricted by HLA-A*26:02 and responses to the C113/114 and C125/126 peptides were restricted by HLA-A*33:03 and HLA-A*02:06, respectively (Fig. 4b).

We next narrowed the optimal epitopes by using overlapping 11-mer and further truncated peptides. Both the C052/053 STCL and C054/055 STCL recognized both Gag 11-121 and Gag 11-122 peptides (Fig. 4c), suggesting that all the STCL were specific for the same epitope. Analysis using truncated peptides showed that the optimal peptide was TLQEQIGWM (TM9) (Fig. 4d) restricted by HLA-A*26:02. This epitope has not been previously reported. The C113/114 STCL and C125/126 STCL recognized Gag 11-200 and Gag 11-214/215, respectively (Fig. 4c). Again using truncated peptides, we identified previously unreported epitopes HIAKNCRAPR (HR10) and RQANFLGKI (RI9) as the optimal peptides presented by HLA-A*33:03 and HLA-A*02:06, respectively (Fig. 4d). Thus, we identified three novel candidate peptides for the Los Alamos National Laboratory HIV Sequence Database (LANL-HSD; www.hiv.lanl.gov) ‘A’ list of well-defined epitopes.

### CD8^+^ T cells recognizing Gag conserved epitopes are protective in vivo

As described in above (Figs. 2b and 3b), T cells specific for 23 reported and 3 novel HIV-1 epitopes recognize tHIVconsvX-derived Gag peptides in HIV-1-infected patients in Japan. To investigate further these T-cell responses, we selected 221 individuals whose PBMCs were available for this analysis. Thus, T cells specific for epitopes SK10/HLA-A*11:01 and KR9/HLA-A*31:01 were not detected in any patients. CD8^+^ T-cells responses recognizing epitopes DT9/HLA-A*24:02, ML8/HLA-A*24:02, NY10/HLA-B*35:01, NL11/HLA-B*67:01, IV9/HLA-A*02, YL9/HLA-A*02:07 and TW10/HLA-B*58:01 were only detected in 1 or 2 patients (Table 1) and were excluded from further statistical analysis because of the small number of responders. The analysis of CD8^+^ T-cell responses to 9 epitopes with sufficient number of responders showed a significant association with lower pVL and/or higher CD4 count relative to non-responders to these epitopes. T cells specific for the WV8/HLA-B*52:01, RI8/HLA-B*52:01 and AA9/HLA-A*02:06 epitopes were previously shown to inhibit HIV-1 in vivo [21, 22]. Therefore, the present study in treatment-naïve, HIV-1-positive patients newly identified T cells specific for 6 epitopes FS8/HLA-A*02:01, TL8/HLA-B*40:02, KL9/HLA-C*08, RI9/HLA-A*02:06, TM9/HLA-A*26:02 and HR10/HLA-A*33:03, that could effectively suppress HIV-1 replication (Table 1).

**Table 1:** Association of CTL responses to Gag epitopes with pVL and CD4 count

Epitope	Sequence	HLA	Frequency		Median of pVL		Median of CD4		P value^b^	
			Res	Non-res	Res	Non-res	Res	Non-res	pVL	CD4
IV9	IILGLNKIV	A*02	2	219	–	–	–	–	–	–
FS8	FLGKIWPS	A*02:01	3	218	910,000	55,500	629	274	0.9882	*0.012*
AA9	ATLEEMMTA	A*02:06	16	205	22,500	58,000	388	269	*0.0057*	0.1788
YL9	YVDRFYKTL	A*02:07	2	219	–	–	–	–	–	–
SK10	SILDIKQGPK	A*11:01	0	221	–	–	–	–	–	–
AK11	ACQGVGGPGHK	A*11:01	4	217	36,500	56,000	450	274	0.3586	0.1277
DT9	DYVDRFYKT	A*24:02	1	220	–	–	–	–	–	–
ML8	MYSPTSIL	A*24:02	1	220	–	–	–	–	–	–
KR9	KIWPSHKGR	A*31:01	0	221	–	–	–	–	–	–
IK10	IAKNCRAPRK	A*31:01	9	212	67,000	55,500	325	275	0.4332	0.7564
DR11	DYVDRFYKTLR	A*33:03	5	216	55,000	56,000	185	279	0.5951	0.3866
GL9	GPGHKARVL	B*07:02	3	218	38,000	56,500	646	274	0.402	0.0741
GY9	GLNKIVRMY	B*15:01	13	208	100,000	55,000	269	285	0.6736	0.8588
NY10	NPPIPVGEIY	B*35:01	1	220	–	–	–	–	–	–
TL8	TERQANFL	B*40:02	23	198	45,000	59,500	416	275	0.1029	*0.0392*
WV8	WMTETLLV	B*52:01	22	199	39,500	58,000	405	269	*0.0481*	*0.0006*
RI8	RMYSPTSI	B*52:01	31	190	43,000	59,500	389	264	*0.0414*	*0.0005*
TW10	TSTLQEQIGW	B*58:01	2	219	–	–	–	–	–	–
NL11	NPDCKTILRAL	B*67:01	1	220	–	–	–	–	–	–
YI9	YSPTSILDI	C*01:02	10	211	123,000	55,000	214	281	0.0919	0.159
KL9	KALGPAATL	C*03	9	212	24,000	56,000	307	275	0.368	0.7865
YL9	YVDRFYKTL	C*03	5	216	76,000	55,500	224	285	0.7639	0.6464
KL9	KALGPAATL	C*08	13	208	24,000	58,000	320	274	*0.0228*	0.64
RI9^a^	RQANFLGKI	A*02:06	4	217	15,000	57,000	319	274	*0.009*	0.9741
TM9^a^	TLQEQIGWM	A*26:02	7	214	39,000	57,500	410	274	*0.032*	0.0921
HR10^a^	HIAKNCRAPR	A*33:03	9	212	4900	58,000	416	273	*0.0002*	*0.0196*

^a^New epitope

^b^Statistically analyzed differences in pVL or CD4 count between responders and non-responders by Mann–Whitney test. Italics indicates that differences were statistically significant

**Fig. 5:**
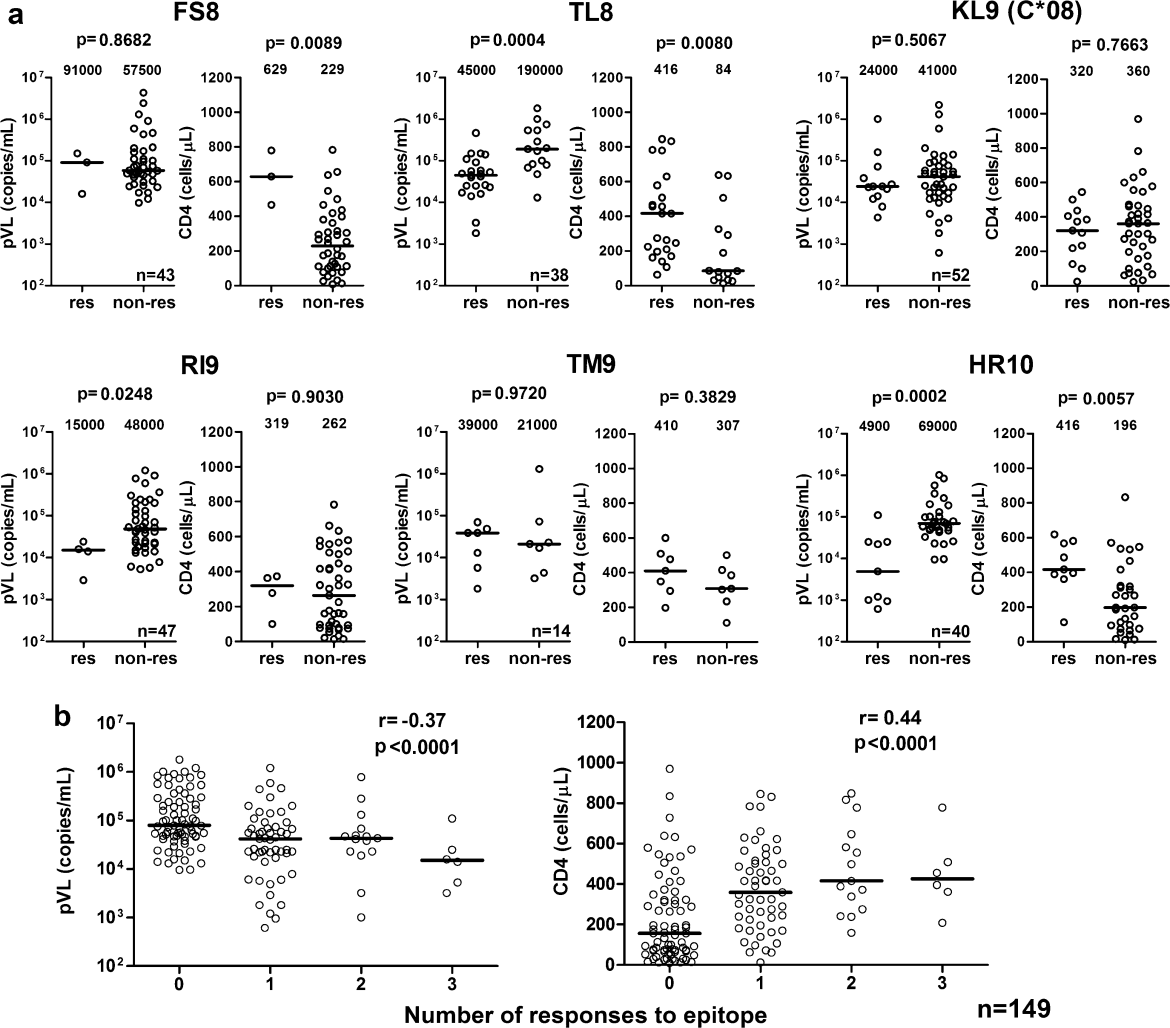
Association of epitope-specific T-cell responses with clinical parameters in HIV-1-infected individuals with the restricting HLA. **a** T-cell responses to 6 epitope peptides were analyzed by using an IFN-γ ELISPOT assay. The differences in pVL or CD4 count between responders and non-responders to each epitope peptide in the individuals having HLA restriction molecules for the epitopes were statistically analyzed by using the Mann-Whitney test. **b** Correlation between a breadth of T-cell responses to 5 epitopes (AA9, TL8, WV8, RI8, and HR10) and pVL and CD4 count in 149 individuals carrying the HLA restriction molecules. The correlation was statistically analyzed using Spearman rank test

We further analyzed the association of responses to these 9 epitopes with lower pVL and/or higher CD4 count in individuals having the epitopes’ restricting HLA molecules. We again confirmed that responders to the WV8/HLA-B*52:01, RI8/HLA-B*52:01 or AA9/HLA-A*02:06 epitopes had significantly lower pVL than non-responders with the same HLA alleles, while responders to WV8 and RI8 also had significantly higher CD4 count than the non-responders (Additional file 2: Fig. S2). Responders to HR10/HLA-A*33:03 and TL8/HLA-B*40:02 had significantly both lower pVL and higher CD4 count than the non-responders (Fig. 5a). Thus overall, responses to epitopes WV8, RI8, AA9, HR10, and TL8 exhibited signs of better clinical outcome. We also analyzed the impact of the total breadth (number of recognized epitopes) and magnitude of the T-cell responses to 5 protective epitopes AA9, TL8, WV8, RI8, and HR10 on clinical outcome and found their very strong negative and positive association with respective pVL (breadth: *p* < 1 × 10^−4^, r = − 0.37; magnitude: *p *< 1 × 10^−4^, r = − 0.40) and CD4 count (breadth: *p* < 1 × 10^−4^, r = 0.44; magnitude: *p* < 1 × 10^−4^, r = 0.44), respectively (Fig. 5b and Additional file 3: Fig. S3).

### TL8- and HR10-specific T cells suppress HIV-1 replication in vitro

**Fig. 6:**
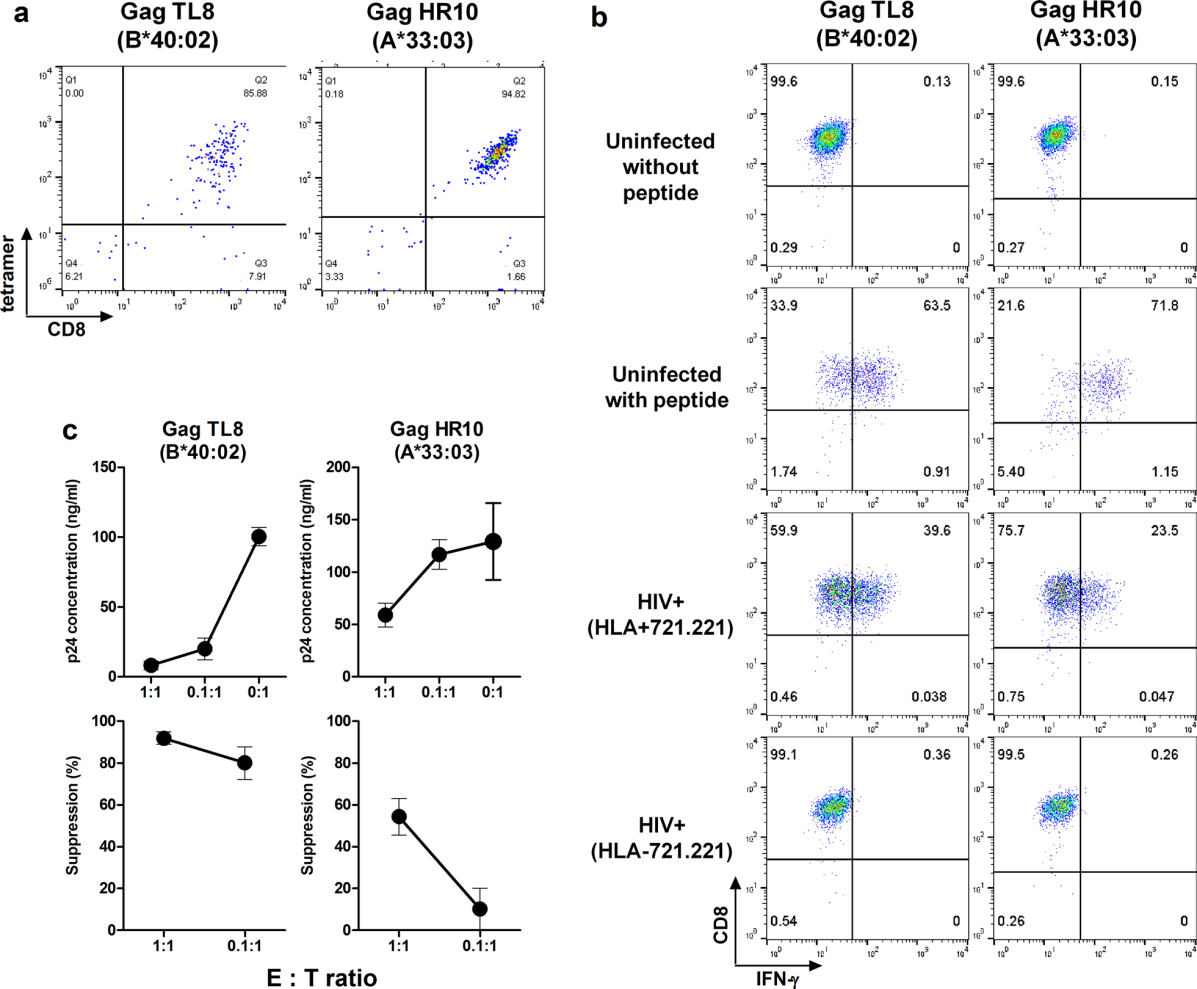
Ability of CTLs to recognize HIV-1-infected cells and to suppress HIV-1 replication in vitro. **a** Generation of the T-cell lines specific for TL8 or HR10 from PBMCs of HLA-B*40:02^+^ individual (KI-1391) or HLA-A*33:03^+^ one (KI-1320). The T-cell lines were established as shown in Materials and Methods. The T-cell lines established were stained with the specific tetramers. **b** Recognition of HIV-1-infected cells by CTLs specific for TL8 or HR10 epitopes. The T-cell lines stimulated with HIV-1 (NL4-3)-infected 721.221 cells (HIV+) expressing the corresponding HLAs or HLA-negative 721.221 cells, and IFN-γ production from the T cells was measured by the ICS assay. The proportions of 721.221-B4002, -A3303 and HLA-negative 721.221 cells infected with NL4-3 were 56.0, 59.7, and 64.1%, respectively. **c** Suppression of HIV-1 replication by the T-cell lines specific for TL8 or HR10. Primary CD4^+^ T-cells from healthy donors carrying the corresponding HLA alleles were infected with NL4-3, and then co-cultured with epitope-specific T cells at E:T ratios of 1:1 and 0.1:1. The concentration of p24 Ag in the culture supernatant was measured by using an enzyme-linked immunosorbent assay. The percentage of suppression was calculated as follows: (concentration of p24 without CTLs – concentration of p24 with CTLs) / concentration of p24 without CTLs × 100. The data are presented as the mean and SD (n = 3)

We next investigated the ability of T cells specific for the reported TL8/HLA-B*40:02 [23, 24] and novel HR10/A*33:03 epitopes to suppress HIV-1 replication *in vitro*. We established T-cell lines specific for TL8 and HR10 from PBMCs of individuals KI-1391 (HLA-B*40:02^+^) and KI-1320 (HLA-A*33:03^+^), respectively, by FACS sorting using the HLA/peptide tetrameric complexes (Fig. 6a). TL8- and HR10-specific T-cell lines were effectively stimulated by peptide-pulsed and HIV-1-infected target cells 721.221/HLA-B*40:02 and 721.221/HLA-A*33:03, respectively, but not uninfected or HLA-untransfected 721.221 cells (Fig. 6b). These T-cell lines efficiently suppressed HIV-1 replication in vitro (Fig. 6c). This HIV-1-inhibitory activity concurs with their improved clinical outcome.

### Gag-specific CD8^+^ T cells cross-recognize epitope variants

**Fig. 7:**
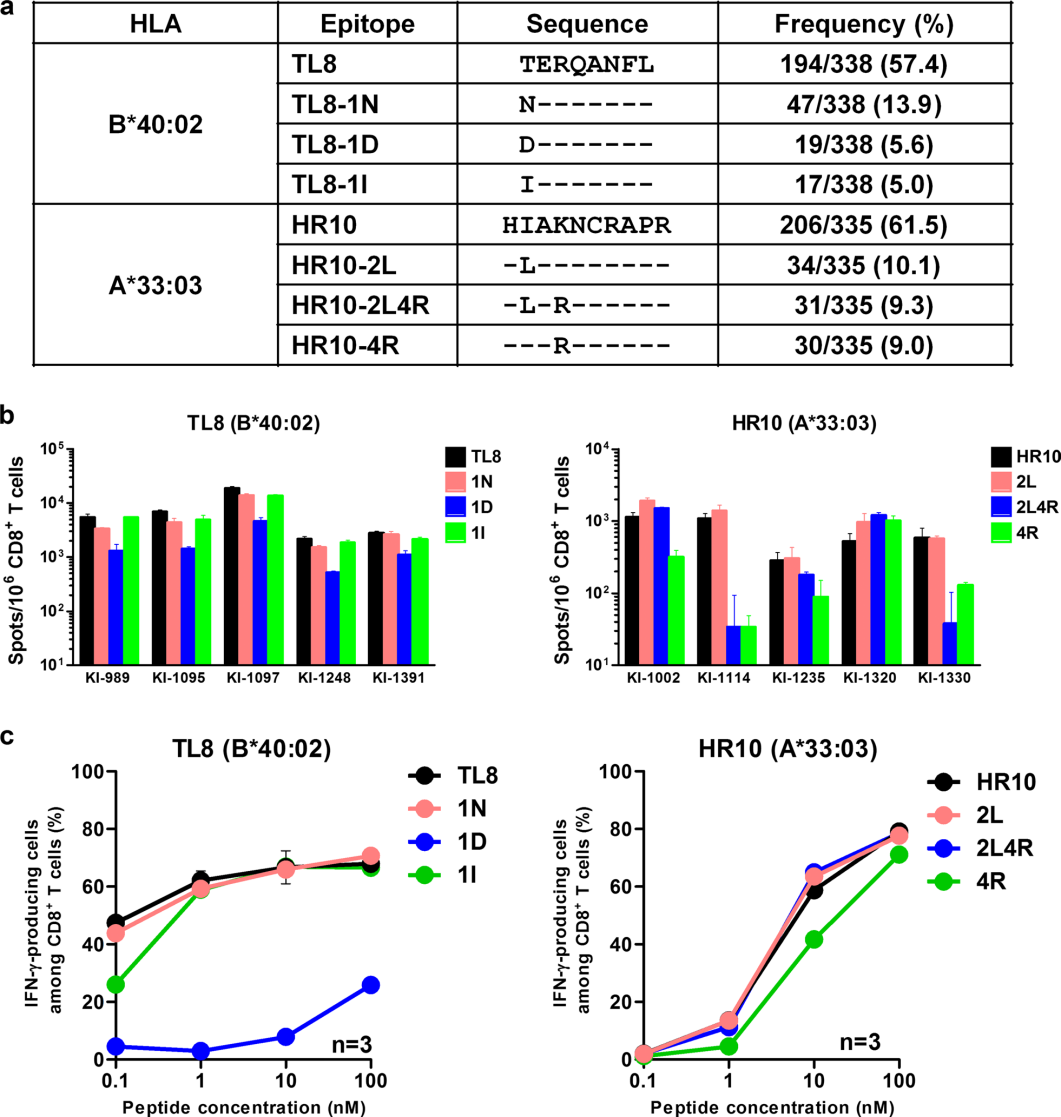
Recognition of mutant epitope peptides by T cells in HIV-1-infected individuals. **a** Frequencies of TL8 and HR10 mutant epitopes in Japanese individuals. The frequencies of mutant epitopes were investigated from 430 chronically HIV-1-infected Japanese individuals. **b** The CD8^+^ T-cell responses to the index and mutant epitope peptides in HIV-1-infected Japanese individuals were analyzed by using an IFN-γ ELISPOT assay. The results are shown as mean and SD (n = 3). **c** The responses of the T cell-lines specific for TL8 or HR10 to wild-type or mutant peptide-prepulsed 721.221 cells expressing the corresponding HLA alleles were analyzed by using the ICS assay. The results are shown as mean and SD (n = 3)

Our previous study showed that CD8^+^ T cells specific for conserved epitopes WV8/HLA-B*52:01 and AA9/HLA-A*02:06 recognized 97 and 90%, respectively, of the circulating viruses in Japan. In contrast, RI8/HLA-B*52:01-specific T cells selected escape mutations, which were present in approximately 27% of the circulating HIV-1 variants [21]. Consensus sequences of HR10/HLA-A*33:03 and TL8/HLA-B*40:02 were found in approximately 60% of circulating viruses in the present Japanese cohort. We therefore analyzed cross-recognition of mutant epitopes by CD8^+^ T cells recognizing the two conserved Gag epitopes, HR10 and TL8. Three mutants for each of these epitopes were detected in > 5% of circulating viruses (Fig. 7a). We therefore assessed the recognition of these mutant epitope peptides by using the IFN-γ ELISPOT assay. Three TL8 mutant peptides were cross-recognized by T cells in 5 HIV-1-infected HLA-B*40:02^+^ patients with the TL8-specific responses, although the recognition of the 1D mutation was reduced (Fig. 7b). Indeed in a dose response analysis, the stimulation of the TL8-specific T-cell line by the 1D mutant peptide was severely reduced (Fig. 7c). The HLA-B*40:02-associated mutation in Gag T427N (p = 1.0 × 10^−9^, *q* = 3.2 × 10^−6^) was found within the TL8/HLA-B*40:02 epitope [25], suggesting that this mutation represented an escape from CD8^+^ T-cell recognition. Nevertheless, the TL8-1N mutated peptide was cross-recognized in all of the 5 tested individuals including 2 having the TL8-1N mutant viruses (Additional file 4: Fig. S4). Similarly, the HR10-2L mutant peptide was cross-recognized by T cells in all 5 HIV-1-infected HLA-A*33:03^+^ individuals who were not infected with HR10-2L or HR10-4R mutant viruses (Additional file 4: Fig. S4), whereas HR10-2L4R and/or HR10-4R mutant peptides were cross-recognized by T cells in patients KI-1002 and KI-1320. HR10-specific T cells from KI-1320 confirmed cross-recognition of these mutant peptides (Fig. 7c). Thus, CD8^+^ T-cells responses specific for TL8 and HR10 epitopes can recognize 70–75% of the circulating HIV-1 isolates in Japan.

## Discussion

Unique vaccine immunogens tHIVconsvX were assembled, which use 2 Gag and 4 Pol conserved regions to focus T-cell responses on the most vulnerable regions (not full-size proteins and not epitopes) of HIV-1 with the bi-valent mosaic design providing the vaccine with a high match to global HIV-1 variants. To date, the immunogenicity of these regions was demonstrated in preclinical studies [14, 26]. Importantly, we were previously able to show that CD8^+^ T-cell responses to 10 peptide pools derived from the two tHIVconsvX mosaic immunogens correlated significantly with low pVL and high CD4 count in 120 treatment-naïve, HIV-1-infected Japanese patients [14]. In the present study, we focused on Gag-specific CD8^+^ T-cells because of their strong contribution to favorable clinical outcome. We defined a number of optimal epitopes and their HLA restriction, which were recognized by CD8^+^ T cells specific for the Gag conserved regions of tHIVconsvX. Furthermore, we demonstrated that 9 Gag-specific CD8^+^ T-cell responses were significantly associated with low pVL and/or high CD4 count in an extended 221-patient cohort of treatment-naïve patients, implying that T cells specific for these 9 epitopes may contribute to suppression of HIV-1 replication in these individuals. To increase the analysis sensitivity and further endorse the ability of these CD8^+^ T-cell responses to suppress HIV-1 replication, we confirmed the association of these T-cells with good clinical parameters among the individuals carrying the appropriate HLA restriction molecules. Finally, we identified 5 Gag epitope-specific protective CD8^+^ T-cell responses and showed a strong association of the magnitude and the breadth of the responses to the 5 epitopes with favorable pVL or CD4 count, indicating again that the responses to 5 Gag epitopes are beneficial. Although escape mutants were selected by the RI8-specific T cells and these HIV-1 mutants accumulated in the Japanese population [21], 70% of circulating HIV-1 still contain variants of the RI8 epitope recognized by RI8-elicited T cells.

It has now been well documented that not all CD8^+^ T-cell responses contribute to the suppression of HIV-1 replication equally [14, 15, 21, 27]. Mothe et al. showed that the T-cell responses to forty-eight 18-mer overlapping peptides associated with low viral load in South Africa, Peru, and Spain cohorts [27]. These 18-mer overlapping peptides covering Gag, Pol, Nef, and Vif regions include 59 optimal defined epitopes in the LANL-HSD. The tHIVconsvX immunogen contains most of these; 19 in Gag and 14 in Pol. In the present study, we identified CD8^+^ T cells specific for 5 Gag conserved epitopes with the ability to suppress HIV-1 replication in Japanese individuals. These CD8^+^ T cells are restricted by HLA-B*52:01, HLA-A*02:06, HLA-A*33:03, and HLA-B*40:02, and at least one of these four alleles are shared in approximately 70% of Japanese population [28]. Since at least one of these alleles is also found in 45% of Chinese [29], 35% of Vietnamese [30], 40% of Thai [31], 50% of Indian [32], and 65% of Korean [33], we hypothesize that if indeed effective, the tHIVconsvX vaccine could be deployed in all these countries; the vaccine would be universal.

To confirm whether the 5 Gag epitopes were recognized by specific T cells as conserved epitopes, we analyzed cross-recognitions of mutant epitopes among the subtype B using the IFN-γ ELISPOT assay. Notably, the circulating viruses carry epitopes with a perfect 9/9 amino acid match or cross-recognized variations in 70–97% isolates from the Japanese population and 46–97% of subtype B viruses (LANL-HSD) and were recognized by T cells specific for 4 epitopes WV8, AA9, TL8, and HR10, though escape mutants selected by the RI8-specific T cells were detected in 27% of the circulating virus in the Japanese cohort [21]. These results suggest that the 4 epitopes are functionally conserved at least among the subtype B viruses. We further analyzed HLA-associated mutations within these six epitopes and found HLA-B*40:02-associated Gag mutation T427N within the HLA-B*40:02/TL8 epitope [25], suggesting that this mutant is accumulated in the HLA-B*40:02^+^ Japanese individuals. However, since mutant Gag epitope TL8-1N was cross-recognized by CD8^+^ T cells in 5 individuals with HLA-B*40:02 (Fig. 7b), TL8-1N may elicit a new T-cell repertoire specific for the 1N mutant or cross-recognizing this mutant. CD8^+^ T-cells responses specific for TL8 and HR10 epitopes can recognize 70–75% of the circulating HIV-1 isolates in Japan while those specific for WV8, AA9, and RI8 did recognized > 70% of them.

We previously demonstrated that three HLA-B*52:01-, two HLA-B*67:01-, and one HLA-B*02:06-restricted CD8^+^ T cells specific for Gag epitopes had a strong ability to suppress HIV-1 replication in the Japanese population [21]. The present study demonstrated that CD8^+^ T cells specific for additional 2 Gag epitopes also contribute to the suppression of HIV-1. Although contained in the tHIVconsvX vaccines, T cells specific for MI8 restricted by HLA-B*52:01 [21], and TL9 and NL11 restricted by HLA-B*67:01 [21] were not identified in this study. This may be explained by the fact that MI8 and TL9 are included in Pool 1 and that the HLA-B*67:01 is a rare allele found in only 4 out of 221 patients involved in the present study. These findings suggest that Japanese individuals vaccinated with the tHIVconsvX vaccines may respond to 8 Gag epitopes and suppress better HIV-1 replication by killer T cells (Additional file 5: Fig. S5).

The present work supports the hypothesis that the CD8^+^ T-cell responses, which the tHIVconsvX vaccines aim to elicit, have a real chance to contribute to the suppression of HIV-1 replication *in vivo* in Japan and other Asian populations both in the context of prevention of HIV-1 infection complementing neutralizing antibodies and in HIV cure by eradicating latently infected cells after HIV-1 reactivation. These results warrantee timely testing of this target immunogen strategy in the clinic.

## Methods

### Subjects

All treatment-naïve Japanese individuals chronically infected with HIV-1 subtype B were recruited from the National Center for Global Health and Medicine. This study was approved by the ethics committees of Kumamoto University and the National Center for Global Health and Medicine. Informed consent was obtained from all individuals according to the Declaration of Helsinki. Plasma and PBMCs were separated from whole blood. HLA types of the individuals were determined by standard sequence-based genotyping.

### Peptides

The mosaic proteins maximize the coverage of potential T-cell epitopes for the global circulating viruses. We generated three pools containing pairs of 15-mer Gag peptides overlapped by 11 amino acids covering two mosaic regions in the tHIVconsvX. Each pool contains 17 to 23 pairs of the 15-mer peptides. Pool 1, 2, and 3 cover Gag 133-231, Gag 221-327, and Gag 317-363/391-459, respectively [14]. The sequences of the 15-mer peptides in each Pool are shown in Additional file 6: Fig. S6. The 15-mer peptides derived from the tHIVconsvX mosaics were generously provided by the International AIDS Vaccine Initiative. Shorter mapping peptides were synthesized by utilizing an automated multiple peptide synthesizer and purified by high-performance liquid chromatography (HPLC). Purity of all peptides (> 90%) was examined by HPLC and mass spectrometry.

### Cell lines

C1R cells expressing HLA-A*26:01 (C1R-A2601), HLA-A*26:02 (C1R-A2602), HLA-A*33:03 (C1R-A3303), HLA-B*07:02 (C1R-B0702), HLA-B*15:01 (C1R-B1501), HLA-B*39:01 (C1R-B3901), or HLA-B*44:03 (C1R-B4403) were previously generated by transfecting these genes into C1R cell lines [21, 34–39]. 721.221 cells expressing CD4 molecules and HLA-C*03:03 (721.221-C0303) were generated by transfecting the genes into the 721.221 cell line. 721.221 cells expressing CD4 molecules and HLA-A*02:06 (721.221-A0206), HLA-A*24:02 (721.221-A2402), HLA-A*33:03 (721.221-A3303), HLA-B*40:02 (721.221-B4002), HLA-C*14:03 (721.221-C1403), or HLA-C*07:02 (721.221-C0702) were previously generated [21, 40–43]. All cell lines were cultured in RPMI 1640 medium containing 10% FCS medium (R10) with 0.15 mg/ml hygromycin B.

### Expansion of HIV-1-specific T cells from HIV-1-infected individuals

PBMCs from KI-1020, KI-1102, and KI-1114 were incubated with 1 μM 15-mer peptide pairs (C052/053 or C054/055, C113/114, and C125/126, respectively) and cultured for 12-14 days to induce peptide-specific short-term cell lines (STCL).

### Intracellular cytokine staining (ICS) assay

C1R and 721.221 cells prepulsed with each peptide or 721.221 cells infected with the HIV-1, strain NL4-3, were added to the effector STCLs in a 96-well plate and incubated for 2 h at 37 °C. Brefeldin A (10 μg/ml) was then added and the cells were incubated further for 4 h, fixed with 4% paraformaldehyde and incubated in permeabilization buffer [0.1% saponin–10% FBS–phosphate-buffered saline (PBS)] after staining with allophycocyanin (APC)-labeled anti-CD8 monoclonal antibody (mAb) (Dako, Glostrup, Denmark). Thereafter, the cells were stained with fluorescein isothiocyanate (FITC)-labeled anti-interferon γ (IFN-γ) mAb (BD Bioscience, CA). The percentage of IFN-γ-producing cells among the CD8^+^ T-cell population was determined by flow cytometry.

### IFN-γ enzyme-linked immunospot (ELISPOT) assay

1×10^5^ PBMCs from HIV-1-positive individuals and peptides were added to 96-well polyvinylidene plates (Millipore, Bedford, MA) that had been precoated with 5 μg/ml anti-IFN-γ mAb; 1-D1K (Mabtech, Stockholm, Sweden). Peptide pools or 15-mer peptide pairs were used at a concentration of 1 µM whereas optimal epitope peptides at a concentration of 100 nM in this assay. The plates were then incubated for 16 h at 37 °C before the addition of biotinylated anti-IFN-γ mAb (Mabtech) at 1 μg/ml at room temperature for 90 min, streptavidin-conjugated alkaline phosphatase (Mabtech) for at room temperature 60 min. Individual cytokine-producing cells were visualized as dark spots after a 20-min reaction with 5-bromo-4-chloro-3-indolyl phosphate and nitro blue tetrazolium in the presence of an alkaline phosphatase-conjugated substrate (Bio-Rad, Richmond, CA, USA). The spots were counted with an Eliphoto-Counter (Minerva Teck, Tokyo, Japan). The frequencies of the responding cells were represented as spot-forming units (SFU)/10^6^ CD8^+^ T cells by measuring frequency of CD8^+^ T cells using a flow cytometry. A mean+5 SD of the SFUs of samples (N = 3) from 12 HIV-1-naïve individuals for the peptide pool was 115 SFU/10^6^ CD8^+^ T cells. Therefore, we defined a positive ELISPOT response as larger than 200 SFU/10^6^ CD8^+^ T cells to exclude false positive.

### Establishment of T-cell lines specific for TL8 and HR10 peptides using HLA/peptide tetramer complexes

To establish T-cell lines specific for TL8 and HR10 peptides, HLA-B*40:02/TL8 or HLA-A*33:03/HR10 tetrameric complexes (tetramers) were synthesized as previously described [44]. Briefly, PBMCs of HLA-B*40:02^+^ individual (KI-1391) and HLA-A*33:03^+^ one (KI-1320) were stained with PE-conjugated specific tetramers at a concentration of 100 nM at 37 °C for 30 min. The cells were then washed twice with R10, followed by staining with FITC-conjugated anti-CD8 mAb and 7-AAD at 4 °C for 30 min. The CD8^+^ T cells specific for the TL8 and HR10 peptides were then sorted by the FACSAria. The sorted cells were stimulated with 100 nM of the corresponding epitope peptides and then cultured for 12–14 days to induce T cell lines specific for TL8 and HR10. To confirm purities of the specific T cells, the cell lines were analyzed by using the specific tetramers.

### In vitro virus inhibition assay

The ability of HIV-1-specific CTLs to suppress HIV-1 replication was examined as previously described [45, 46]. CD4^+^ T cells isolated from PBMCs of healthy donors carrying HLA-B*40:02 or -A*33:03 were infected with NL4-3 and then the infected cells were co-cultured with epitope-specific T-cell lines at E:T ratios of 1:1 and 0.1:1. On day 3–4 post infection, the concentration of p24 Ag in the culture supernatant was measured by using an enzyme-linked immunosorbent assay.

### Statistical analyses

Two-tailed Mann–Whitney’s test was performed for comparison of two groups. Correlations between magnitudes and breadths of T cell responses and pVL or CD4 count were statistically analyzed using Spearman rank test. P values < 0.05 were considered to be statistically significant.

## Additional files

**Additional file 1: Fig. S1.** Correlation of the magnitudes of the Gag responses with pVL and CD4 count. T-cell responses to Gag peptide Pools 1, 2 and 3 derived from vaccine immunogen tHIVconsvX were enumerated using an IFN-γ ELISPOT assay in 200 HIV-1-infected Japanese individuals. Correlation coefficients (r) and p-values were determined by using the Spearman rank correlation test.

**Additional file 2: Fig. S2.** Association of the T-cell responses to AA9, WV8, or RI8 with pVL or CD4 count. T-cell responses to the 3 epitope peptides were analyzed by using the IFN-γ ELISPOT assay. The differences in pVL or CD4 count between responders and non-responders to each epitope peptide in the individuals having HLA restriction molecules for the epitopes were statistically analyzed by using the Mann-Whitney test.

**Additional file 3: Fig. S3.** Correlation between a total magnitude of T-cell responses to 5 epitopes and pVL and CD4 count. T-cell responses to 5 epitope peptides (AA9, TL8, WV8, RI8, and HR10) were analyzed in 149 individuals carrying the HLA restriction molecules by using the IFN-γ ELISPOT assay. Correlation coefficients (r) and p-values were determined by using the Spearman rank correlation test.

**Additional file 4: Fig. S4.** HIV-1 sequences within Gag TL8 and Gag HR10 epitopes in HIV-1-infected individuals. HIV-1 sequences within Gag TL8 and Gag HR10 were analyzed in HIV-1-infected individuals tested in Figure 7b. Mutant positions are highlighted in red.

**Additional file 5: Fig. S5.** Location of the 8 Gag CTL epitopes in the tHIVconsvX. The tHIVconsvX vaccine is composed of 2 Gag and 4 Pol conserved fragments. The two complementing mosaic immunogens corresponding to the 6 conserved regions are used in this vaccine. HLA-B*67:01-restricted TL9-specific, HLA-B*52:01-restricted MI8-specific, and HLA-B*67:01-restricted NL11-specific CTLs also have strong abilities to suppress HIV-1 replication in vivo (highlighted in green, Murakoshi et al., 2015).

**Additional file 6: Fig. S6.** List of 15-mer overlapping peptide pairs in Pools 1-3. Pool 1, 2, and 3 cover Gag133-231, Gag221-327, and Gag317-363 / 391-459, respectively.
