# Supplementary figures and images for: CD8+ T cells specific for conserved, cross-reactive Gag epitopes with strong ability to suppress HIV-1 replication
Source: Retrovirology. 2018 Jul 3;15:46. doi: 10.1186/s12977-018-0429-y (PMC6029025; doi:10.1186/s12977-018-0429-y)

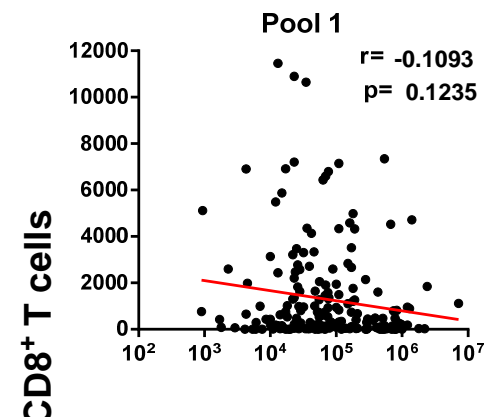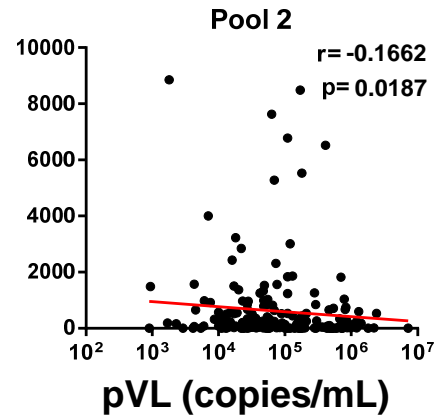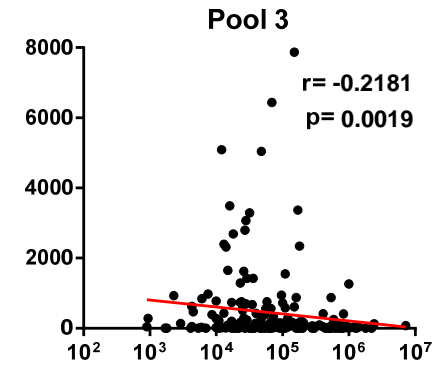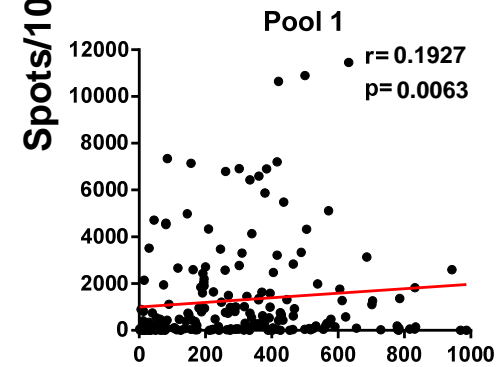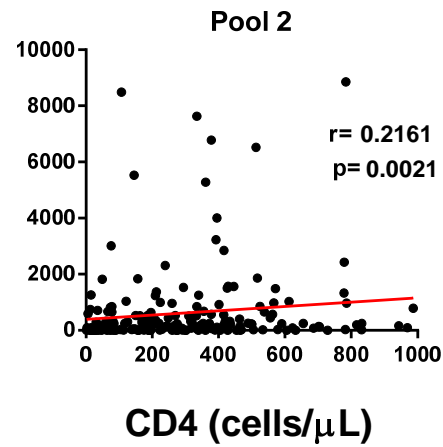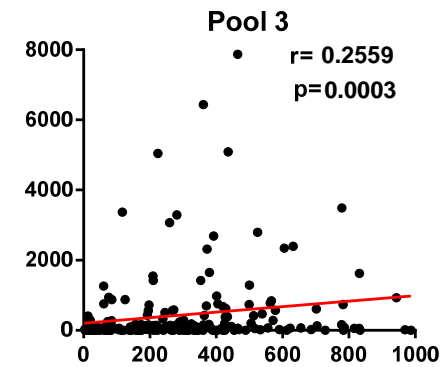

**N=200**

Supplement: Supplementary file 1 — Additional file 1: Fig. S1. Correlation of the magnitudes of the Gag responses with pVL and CD4 count. T-cell responses to Gag peptide Pools 1, 2 and 3 derived from vaccine immunogen tHIVconsvX were enumerated using an IFN-γ ELISPOT assay in 200 HIV-1-infected Japanese individuals. Correlation coefficients (r) and p-values were determined by using the Spearman rank correlation test. [file 12977_2018_429_MOESM1_ESM.pdf]

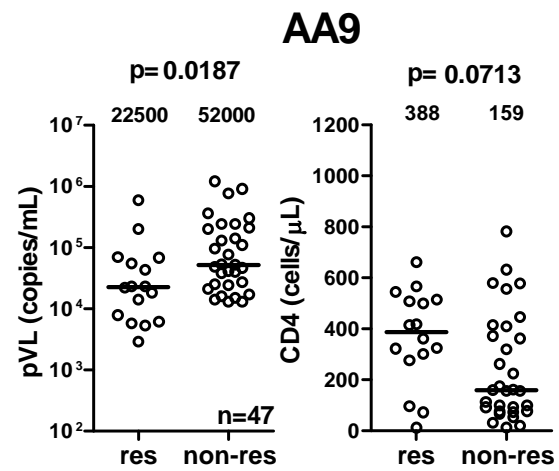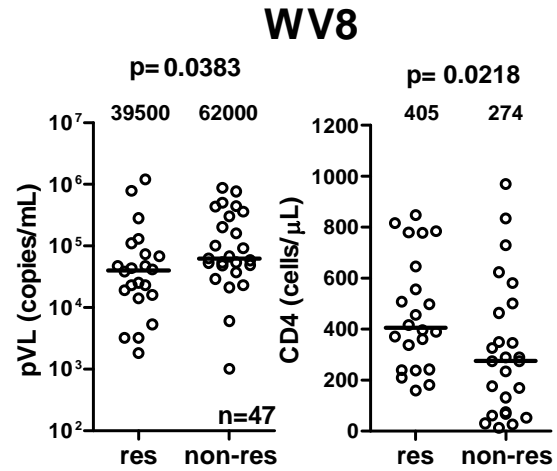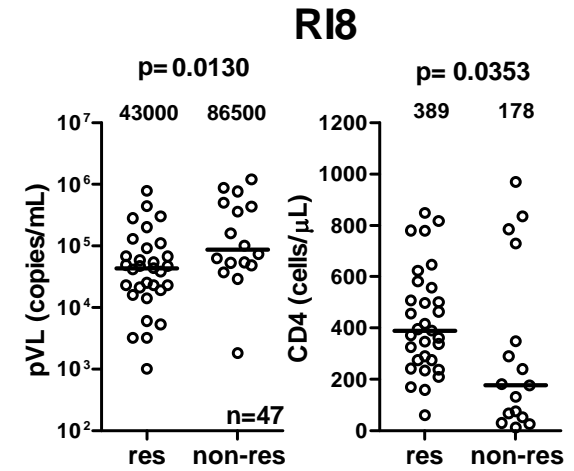

Supplement: Supplementary file 2 — Additional file 2: Fig. S2. Association of the T-cell responses to AA9, WV8, or RI8 with pVL or CD4 count. T-cell responses to the 3 epitope peptides were analyzed by using the IFN-γ ELISPOT assay. The differences in pVL or CD4 count between responders and non-responders to each epitope peptide in the individuals having HLA restriction molecules for the epitopes were statistically analyzed by using the Mann-Whitney test. [file 12977_2018_429_MOESM2_ESM.pdf]

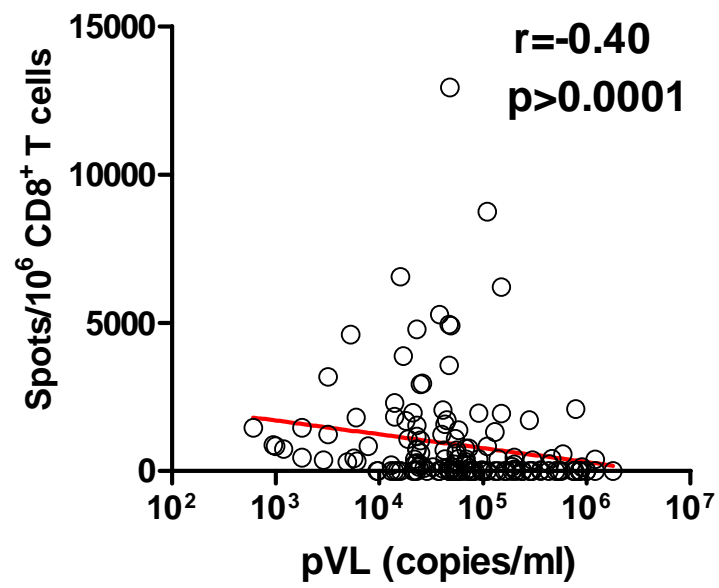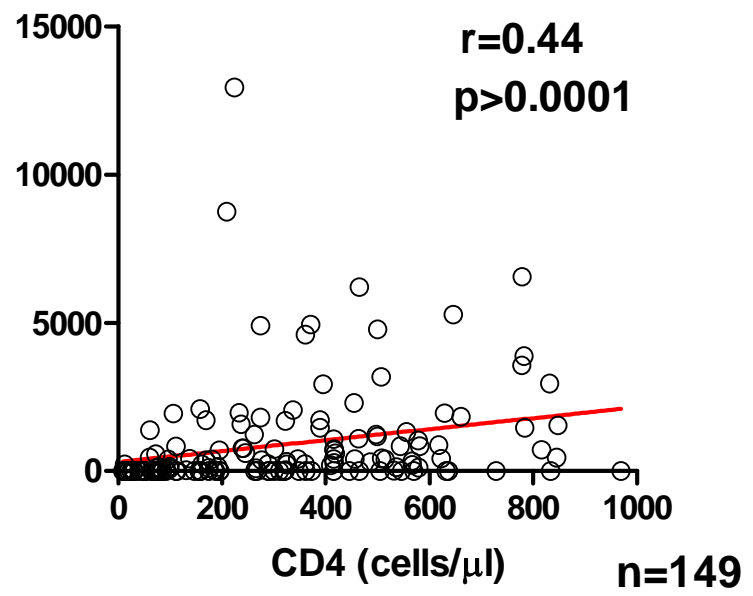

Supplement: Supplementary file 3 — Additional file 3: Fig. S3. Correlation between a total magnitude of T-cell responses to 5 epitopes and pVL and CD4 count. T-cell responses to 5 epitope peptides (AA9, TL8, WV8, RI8, and HR10) were analyzed in 149 individuals carrying the HLA restriction molecules by using the IFN-γ ELISPOT assay. Correlation coefficients (r) and p-values were determined by using the Spearman rank correlation test. [file 12977_2018_429_MOESM3_ESM.pdf]
