# Supplementary material for: CD8+ T cells specific for conserved, cross-reactive Gag epitopes with strong ability to suppress HIV-1 replication
Source: Retrovirology. 2018 Jul 3;15:46. doi: 10.1186/s12977-018-0429-y (PMC6029025; doi:10.1186/s12977-018-0429-y)
Supplement: Supplementary file 4 — Additional file 4: Fig. S4. HIV-1 sequences within Gag TL8 and Gag HR10 epitopes in HIV-1-infected individuals. HIV-1 sequences within Gag TL8 and Gag HR10 were analyzed in HIV-1-infected individuals tested in Figure 7b. Mutant positions are highlighted in red. [file 12977_2018_429_MOESM4_ESM.pdf]

Gag TL8 (B*40:02)

ID	Epitope sequence
KI-989	**TERQANFL**
KI-1095	**TEKQANFL**
KI-1097	**TERQANFL**
KI-1248	**NERQANFL**
KI-1391	**NERQANFL**

Gag HR10 (A*33:03)

ID	Epitope sequence
KI-1002	**HIAKHCRAPR**
KI-1114	**HIAKHCRAPR**
KI-1235	**HIAKNCRAPR**
KI-1320	**HIAKNCRAPR**
KI-1330	**HIAKNCRAPR**
