# Supplementary material for: CD8+ T cells specific for conserved, cross-reactive Gag epitopes with strong ability to suppress HIV-1 replication
Source: Retrovirology. 2018 Jul 3;15:46. doi: 10.1186/s12977-018-0429-y (PMC6029025; doi:10.1186/s12977-018-0429-y)
Supplement: Supplementary file 5 — Additional file 5: Fig. S5. Location of the 8 Gag CTL epitopes in the tHIVconsvX. The tHIVconsvX vaccine is composed of 2 Gag and 4 Pol conserved fragments. The two complementing mosaic immunogens corresponding to the 6 conserved regions are used in this vaccine. HLA-B*67:01-restricted TL9-specific, HLA-B*52:01-restricted MI8-specific, and HLA-B*67:01-restricted NL11-specific CTLs also have strong abilities to suppress HIV-1 replication in vivo (highlighted in green, Murakoshi et al., 2015). [file 12977_2018_429_MOESM5_ESM.pdf]

6 highly functionally conserved regions

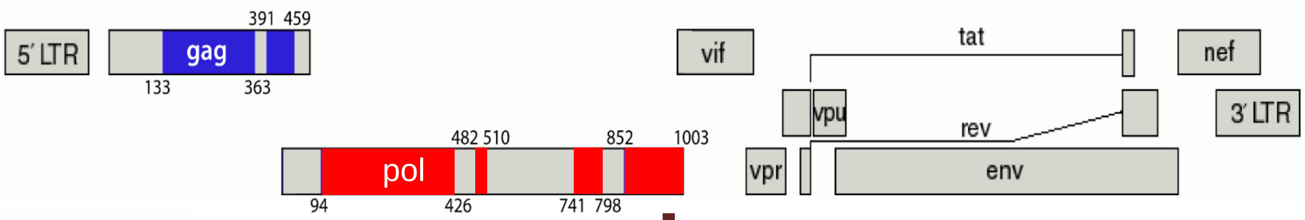

Computed into bi-valent mosaic (always used together)

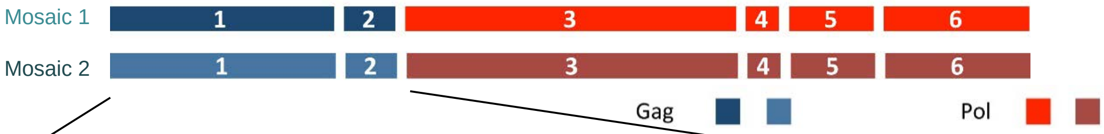

Differ in10% aa

Gag 1 (Mosaic 1)

TL9
PIVQNLQGQMVHQAISPRTLNAWVKVIEEKAFSPEVIPMFTALSEGA**TPQDLNTML**NTVGGHQAAMQMLKDTINEEAAEW
DRVHPVHAGPIAPGQMREPRGSDIAGTTSNLQEQIGWMTSNPPIPVGDIYKRWIILGLNKIVRMYSPVSILDIRQGPKEP
FRDYVDRFFKTLRAEQATQEVKNWMTDTLLVQNANPDCKTILRALGPGATLEEMMTACQGVGGPGHKARVL

Gag 1 (Mosaic 2)

MI8
PIVQNAQGQMVHQALSPRTLNAWVKVVEEKAFSPEVIPMFSALSEGATPQDLNMMLNIVGGHQAA**MQMLKETI**NEEAAEW

RI8
DRLHPVHAGPIPPGQMREPRGSDIAGTTSTLQEQIGWMTNNPPIPVGEIYKRWIIMGLNKIV**RMYSPTSI**LDIKQGPKEP

WV8 NL11 AA9
FRDYVDRFYKTLRAEQASQEVKN**WMTETLLV**QNA**NPDCKTILKAL**GPA**ATLEEMMTA**CQGVGGPSHKARVL

Gag 2 (Mosaic 1)

HR10
KCFNCGKEG**HIAKNCRAPR**KRGCWKCGREGHQMKDCNERQANFLGKIWPSHKGRPGNFLQSRPEPTAPP

Gag 2 (Mosaic 2)

TL8
KCFNCGKEGHLARNCRAPRKKGCWKCGKEGHQMKDC**TERQANFL**GKIWPSNKGRPGNFPQSRPEPSAPP
