# Supplementary material for: CD8+ T cells specific for conserved, cross-reactive Gag epitopes with strong ability to suppress HIV-1 replication
Source: Retrovirology. 2018 Jul 3;15:46. doi: 10.1186/s12977-018-0429-y (PMC6029025; doi:10.1186/s12977-018-0429-y)
Supplement: Supplementary file 6 — Additional file 6: Fig. S6. List of 15-mer overlapping peptide pairs in Pools 1-3. Pool 1, 2, and 3 cover Gag133-231, Gag221-327, and Gag317-363 / 391-459, respectively. [file 12977_2018_429_MOESM6_ESM.pdf]

Pool 1

C001	PIVQNLQGQMVHQAI
C002	PIVQNAQGQMVHQAL
C003	NLQGQMVHQAISPRT
C004	NAQGQMVHQALSPRT
C005	QMVHQAISPRTLNAW
C006	QMVHQALSPRTLNAW
C007	QAISPRTLNAWVKVI
C008	QALSPRTLNAWVKVV
C009	PRTLNAWVKVIEEKA
C010	PRTLNAWVKVVEEKA
C011	NAWVKVIEEKAFSPE
C012	NAWVKVVEEKAFSPE
C013	KVIEEKAFSPEVIPM
C014	KVVEEKAFSPEVIPM
C015	EKAFSPEVIPMFTAL
C016	EKAFSPEVIPMFSAL
C017	SPEVIPMFTALSEGA
C018	SPEVIPMFSALSEGA
C019	IPMFTALSEGATPQD
C020	IPMFSALSEGATPQD
C021	TALSEGATPQDLNTM
C022	SALSEGATPQDLNMM
C023	EGATPQDLNTMLNTV
C024	EGATPQDLNMMLNIV
C025	PQDLNTMLNTVGGHQ
C026	PQDLNMMLNIVGGHQ
C027	NTMLNTVGGHQAAMQ
C028	NMMLNIVGGHQAAMQ
C029	NTVGGHQAAMQMLKD
C030	NIVGGHQAAMQMLKE
C031	GHQAAMQMLKDTINE
C032	GHQAAMQMLKETINE
C033	AMQMLKDTINEEAAE
C034	AMQMLKETINEEAAE
C035	LKDTINEEAAEWDRV
C036	LKETINEEAAEWDRL
C037	INEEAAEWDRVHPVH
C038	INEEAAEWDRLHPVH
C039	AAEWDRVHPVHAGPI
C040	AAEWDRLHPVHAGPI
C041	DRVHPVHAGPIAPGQ
C042	DRLHPVHAGPIPPGQ
C043	PVHAGPIAPGQMREP
C044	PVHAGPIPPGQMREP

Pool 2

C045	GPIAPGQMREPRGSD
C046	GPIPPGQMREPRGSD
C047	PGQMREPRGSDIAGT
C048	REPRGSDIAGTTSNL
C049	REPRGSDIAGTTSTL
C050	GSDIAGTTSNLQEQI
C051	GSDIAGTTSTLQEQI
C052	AGTTSNLQEQIGWMT
C053	AGTTSTLQEQIGWMT
C054	SNLQEQIGWMTSNPP
C055	STLQEQIGWMTNNPP
C056	EQIGWMTSNPPIPVG
C057	EQIGWMTNNPPIPVG
C058	WMTSNPPIPVGDIYK
C059	WMTNNPPIPVGEIYK
C060	NPPIPVGDIYKRWII
C061	NPPIPVGEIYKRWII
C062	PVGDIYKRWIILGLN
C063	PVGEIYKRWIIMGLN
C064	IYKRWIILGLNKIVR
C065	IYKRWIIMGLNKIVR
C066	WIILGLNKIVRMYSP
C067	WIIMGLNKIVRMYSP
C068	GLNKIVRMYSPVSIL
C069	GLNKIVRMYSPTSIL
C070	IVRMYSPVSILDIRQ
C071	IVRMYSPTSILDIKQ
C072	YSPVSILDIRQGPKE
C073	YSPTSILDIKQGPKE
C074	SILDIRQGPKEPFRD
C075	SILDIKQGPKEPFRD
C076	IRQGPKEPFRDYVDR
C077	IKQGPKEPFRDYVDR
C078	PKEPFRDYVDRFFKT
C079	PKEPFRDYVDRFYKT
C080	FRDYVDRFFKTLRAE
C081	FRDYVDRFYKTLRAE
C082	VDRFFKTLRAEQATQ
C083	VDRFYKTLRAEQASQ
C084	FKTLRAEQATQEVKN
C085	YKTLRAEQASQEVKN
C086	RAEQATQEVKNWMTD
C087	RAEQASQEVKNWMTE
C088	ATQEVKNWMTDTLLV
C089	ASQEVKNWMTETLLV
C090	VKNWMTDTLLVQNAN
C091	VKNWMTETLLVQNAN

Pool 3

C092	MTDTLLVQNANPDCK
C093	MTETLLVQNANPDCK
C094	LLVQNANPDCKTILR
C095	LLVQNANPDCKTILK
C096	NANPDCKTILRALGP
C097	NANPDCKTILKALGP
C098	DCKTILRALGPGATL
C099	DCKTILKALGPAATL
C100	ILRALGPGATLEEMM
C101	ILKALGPAATLEEMM
C102	LGPGATLEEMMTACQ
C103	LGPAATLEEMMTACQ
C104	ATLEEMMTACQGVGG
C105	EMMTACQGVGGPGHK
C106	EMMTACQGVGGPSHK
C107	ACQGVGGPGHKARVL
C108	ACQGVGGPSHKARVL
C109	KCFNCGKEGHIAKNC
C110	KCFNCGKEGHLARNC
C111	CGKEGHIAKNCRAPR
C112	CGKEGHLARNCRAPR
C113	GHIAKNCRAPRKRGC
C114	GHLARNCRAPRKKGC
C115	KNCRAPRKRGCWKCG
C116	RNCRAPRKKGCWKCG
C117	APRKRGCWKCGREGH
C118	APRKKGCWKCGKEGH
C119	RGCWKCGREGHQMKD
C120	KGCWKCGKEGHQMKD
C121	KCGREGHQMKDCNER
C122	KCGKEGHQMKDCTER
C123	EGHQMKDCNERQANF
C124	EGHQMKDCTERQANF
C125	MKDCNERQANFLGKI
C126	MKDCTERQANFLGKI
C127	NERQANFLGKIWPSH
C128	TERQANFLGKIWPSN
C129	ANFLGKIWPSHKGRP
C130	ANFLGKIWPSNKGRP
C131	GKIWPSHKGRPGNFL
C132	GKIWPSNKGRPGNFP
C133	PSHKGRPGNFLQSRP
C134	PSNKGRPGNFPQSRP
C135	GRPGNFLQSRPEPTA
C136	GRPGNFPQSRPEPSA
C137	NFLQSRPEPTAPP
C138	NFPQSRPEPSAPP

Blue : Mosaic 1
Red : Mosaic 2
